# Experimental and Numerical Investigation of the Mechanical Properties of a Fiber-Reinforced Geopolymer Mortar Blast Resistant Panel

**DOI:** 10.3390/polym15163440

**Published:** 2023-08-17

**Authors:** Chien-Chin Chen, Ying-Kuan Tsai, Yu-Kai Lin, Pin-Hsuan Ho, Chang-Yu Kuo

**Affiliations:** 1 Department of Environmental Information and Engineering, Chung Cheng Institute of Technology, National Defense University, Taoyuan 33550, Taiwan; 2 Department of Civil Engineering, National Cheng Kung University, Tainan 70101, Taiwan

**Keywords:** blast resistant panel, geopolymer, carbon fiber, Kevlar fiber, ultra-high performance polyethylene (PE) fiber, free-air explosion, finite element analysis

## Abstract

Geopolymer materials have excellent properties such as high strength, low thermal conductivity, fire resistance, acid and alkali resistance, and low carbon emissions. They can be used as protective engineering materials in places with explosion risks. At present, the common composite blast resistant panel is in the form of a sandwich: the outer layer isgalvanized steel plate, and fiber cement board or calcium carbonate board is used as the inner layer material, as these boards have the advantages of easy installation, good fire resistance, and explosion resistance. This study investigates the effect of adding different types of fibers to geopolymer mortar on the mortar’s basic mechanical properties, such as compression strength, bending strength, and impact resistance. The explosive resistance of the fiber-reinforced geopolymer mortar blast resistant panels was evaluated through free-air explosion. In this paper, experimental procedures and numerical simulation have been performed to study the failure modes, maximum deflection, and dynamic response of the fiber-reinforced geopolymer mortar blast resistant panel under free-air explosion. The research results can provide a reference for the design and production of blast resistant panels.

## 1. Introduction

Nowadays, there has been a rapid growth in the development of green building materials as a response to the need for reducing the environmental impact of construction. The main reason is that the manufacturing process of conventional cementitious materials leads to air pollution and waste generation. In recent years, there has been an increasing inclination towards substituting ordinary Portland cement (OPC) with supplementary or pozzolanic cementitious materials, such as fly ash, in the formulation of mix proportions. This trend is driven by the objective of reducing CO_2_ emissions. Furthermore, the use of alkali-activated cement has emerged as a viable method for effectively replacing OPC in concrete. By reducing the reliance on OPC in cementitious materials, geopolymer has gained significant popularity in the construction industry. Geopolymers offer advantages such as early compressive strength, lower permeability, and enhanced chemical resistance, making it widely applied in construction. Moreover, geopolymer materials possess outstanding properties including low thermal conductivity, fire resistance, and resistance to acids and alkalis [1,2,3,4,5]. These properties make geopolymers highly suitable as engineering materials for protective purposes.

Furthermore, a sandwich structure is an innovative composite system that offers exceptional energy dissipation capability [6,7,8]. Due to these properties, it can effectively serve as an energy absorber in various structures that involve extreme loading conditions, such as ballistic impacts and blast loads. Shock wave load resistance is a vital factor in numerous engineering constructions, and it must be managed within certain actual constraints, such as limits on force transmission or deformation. On the other hand, if significant distortions are acceptable, a limit can be imposed on the force transmitted. The target can be realized by using sandwich structures that dissipate energy through both local crushing of the inner material and bending and stretching of the facings [9,10,11,12]. With a thoughtful choice of the material and thickness of the sandwich structure, it is crucial to design for a specific peak force transmission and the amount of energy that can be efficiently absorbed per unit area. In addition, geopolymer materials exhibit extraordinary mechanical properties and material features, making them an appropriate choice as protective engineering materials in environments exposed to potential explosion risks. Currently, the typical composite blast resistant panel is configured in the form of a sandwich structure. It features an outer layer of galvanized steel plate and uses cementitious material as the inner layer material. This configuration offers benefits such as easy installation, excellent fire resistance, and robust explosion resistance.

Geopolymer materials commonly employ raw materials such as blast furnace slag and silica fume. In recent years, the rise in steel production has led to an increase in the amount of furnace slag being produced, which has had a greater environmental impact [13]. The reaction mechanism of geopolymers is an inorganic polycondensation reaction that yields a three-dimensional aluminosilicate structure with a chemical formula expressed as: $M_{n}{\left[-{\left(S_{i}O_{2}\right)}_{z}-AlO_{2}\right]}_{n}\cdot wH_{2}O$, where *M* represents the cation (K^+^, Na^+^, Ca^2+^), *n* represents the degree of polycondensation, and *z* can be 1, 2, 3, or greater [14,15,16,17]. The inorganic polycondensation reaction mentioned above does not produce CO_2_ emissions.

The primary challenges with geopolymers in certain conditions include a decrease in strength and a rapid expansion of cracks under tension. The toughness and bending resistance of geopolymers can be enhanced by adding fibers, which provide frictional force and establish chemical bonds between the fiber and the geopolymer material [18,19,20,21]. Under intense dynamic loads like blasts and impacts, the geopolymer material may undergo stress wave propagation that can lead to concrete failure, resulting in damage, the formation of craters, spalling, and breaches. These local failure types are difficult to evaluate accurately. A few empirical formulae provided in UFC 3-340-02, the U.S. Department of Defense Unified Facilities Criteria for “Structures to Resist the Effects of Accidental Explosions”, can be applied for predicting the size and diameter of concrete cratering and spalling [22]. However, these formulae are based on experimental results of traditional concrete material, the accuracy still has room to improve, and finite element analysis with validated material parameters is an effective way to achieve this improvement. Numerical simulations are vital in estimating how structural concrete responds and potentially fails when subjected to transient loads such as blast and impact loads. These lead to extreme stress, rapid strain rates, and significant deformations of the target. Owing to the complex responses of brittle materials under dynamic loads, various constitutive material models are accessible in finite element analysis software such as LS-DYNA.

The size of the elements used in Finite Element Analysis (FEA) significantly affects the failure mechanism of structures, especially in the case of ductile fracture in reinforced concrete. Studies of the sensitivity of critical failure strain, damage patterns, and orientations to mesh size in the FEA of geopolymer concrete under transient loads are still limited. The validated material mechanical properties and element size play a crucial role in accurately capturing the cracking pattern of reinforced geopolymer concrete beams. Generally, a finer mesh offers better agreement with the experimental results compared to the coarse mesh, and accurately traces the ductile failure of the structural element [23,24,25].

The concrete structural element is prone to failure brittlely under blast loads. To enhance the protective capacity of a structure, a sandwich composite blast resistant panel with outer layers made of galvanized steel plate and an inner layer made of fiber-reinforced geopolymer mortar is developed and validated experimentally and numerically in this study. Although geopolymer material in previous studies shows better mechanical performance than OPC, it tends to fail brittlely and possesses rapid crack growth in tension when subjected to blast loads. Thus, carbon fiber, Kevlar fiber, and PE fiber were added to the geopolymer mortar to enhance the blast resistant performance. This study investigated the mechanical behavior of fiber-reinforced geopolymer mortar under both static and dynamic loading conditions. Subsequently, field explosion tests were conducted and then analyzed numerically using LS-DYNA (Version R11.1.0).

## 2. Experimental Study

### 2.1. Material

In this research, ground granulated blast furnace slag (GGBS), wollastonite, borax, and silica fume are utilized as raw materials to manufacture a geopolymer, with their chemical compositions detailed in Table 1. GGBS is provided by CHC RESOURCES Co., Ltd. (Kaohsiung, Taiwan). With particle size D_50_, this is 12 μm and conforms to ASTM C989. Wollastonite is provided by Charles Star Enterprise Co., Ltd. (Taipei, Taiwan). With particle size D_50_, this is 44 μm. Silica fume is provided by National Blue Star (Group) Co., Ltd. (Beijing, China), with the size of 80% of the particles being smaller than 1 μm.

**Figure 1:**
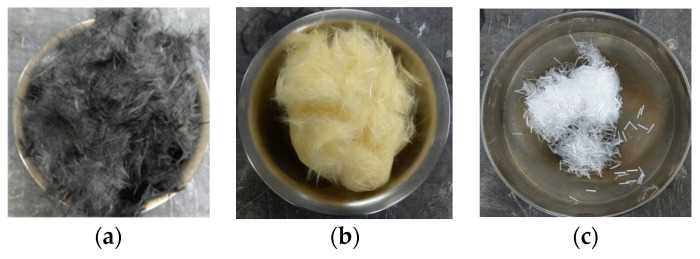
Appearance of fibers: (**a**) carbon fiber; (**b**) Kevlar fiber; (**c**) ultra-high performance PE Fiber.

This study seeks to improve the mechanical performance of geopolymers and reduce the rapid tensile crack expansion by incorporating various types of fibers into the cementitious materials, including carbon fiber, Kevlar fiber, and ultra-high performance polyethylene (PE) fiber, respectively. The carbon fiber employed in this experiment is PAN-based and produced by the Tairylan Division of Formosa Plastics Co., Ltd. (Kaohsiung, Taiwan). Because of the presence of a silane coupling agent in the manufacturing process of carbon fiber, the present study utilized a surface treatment of heating the carbon fiber using the high-temperature approach. The aramid fiber applied in this study is Kevlar^®^ which is manufactured by DuPont Company (Wilmington, DE, USA). The ultra-high performance polyethylene (PE) fiber was supplied by TOYOBO Co., Ltd. (Ōsaka shi, Tokyo). In this study, all types of fibers used were of a consistent length of 12 mm. To enhance fiber distribution within the cementitious materials, the pneumatic dispersion process was used. Figure 1 illustrates the appearances of all fibers used in this study. To enhance the impact-resistant performance of geopolymer mortar, carbon fibers were added due to their excellent tensile strength and tensile modulus. The experiment design added 12 mm carbon fibers with a total weight of 1 g. The weight ratio is 1% compared with the total weight of the GGBS. In addition, the energy absorption capacity plays an important role against impact and blast loads, which is associated with its deformation limit. The Kevlar fiber and ultra-high performance fiber were designed to replace 50% weight of carbon fibers for their better breaking elongation compared to carbon fiber. The physical properties of the three different types of fibers are listed in Table 2.

The geopolymer was prepared using the pre-mixed method with added dispersed fiber to improve mechanical performance. The experimental formulation utilized in this investigation encompassed a geopolymer matrix comprising silica fume and GGBS in a weight ratio of 1:100. The liquid-to-binder ratio and sand-to-binder ratio were established at 0.55 and 1.2, respectively. The alkaline activating solution employed in this research was composed of tap water, industrial-grade NaOH (with a purity of 95%), Na_2_SiO_3_ (characterized by a Baumé scale reading of 15°, Na_2_O content ranging from 9% to 10%, and SiO_2_ content ranging from 28% to 30%), and NaAlO_2_ (with a molar ratio of Na/Al at 1.18, Na_2_O content of 19.5%, and Al_2_O_3_ content of 26.5%). The alkaline activating solution was prepared to yield a molarity of 10 M for NaOH. Notably, the molar ratios of Si/Na and Si/Al were defined as 1.28 and 50, respectively. The abbreviation of the geopolymer material specimens and the detailed mix proportion of the fiber-reinforced geopolymer mortar are shown in Table 3 and Table 4.

### 2.2. Experimental Procedure

The primary objective of this research is to investigate the mechanical properties and blast resistance capacity of fiber-reinforced geopolymer mortar blast resistant panels. To achieve this goal, the workability and shrinkage characteristics of fiber-reinforced geopolymers with the incorporation of various types and quantities of fibers into geopolymer mortar were explored. The preliminary research findings of this study reveal that the addition of silica fume effectively enhances the mechanical properties of the material, leading to the efficient filling of pores within the specimen. This reduction in porosity and water absorption subsequently contributes to an increase in compressive strength. The use of a retarding agent, borax, along with adjustments to the moisture content of the fine aggregate, achieves a retarding effect, further enhancing workability. The incorporation of different fiber types and combinations has shown promise in enhancing the shrinkage behavior of a geopolymer while also influencing its workability. To comprehensively assess the impact of composite fiber addition, a series of both quasi-static and dynamic mechanical tests were conducted. Subsequently, the appropriate mixture was utilized as an internal filling material for the blast resistant panels, which were further coated externally with galvanized steel plates. For additional reinforcement, helical iron wires were incorporated internally to create fiber-reinforced geopolymer composite materials within the blast resistant panels. Finally, the panels were subjected to rigorous explosion impact tests to thoroughly evaluate their suitability and applicability for the intended purpose.

#### 2.2.1. Geopolymer Mechanical Investigation

**Figure 2:**
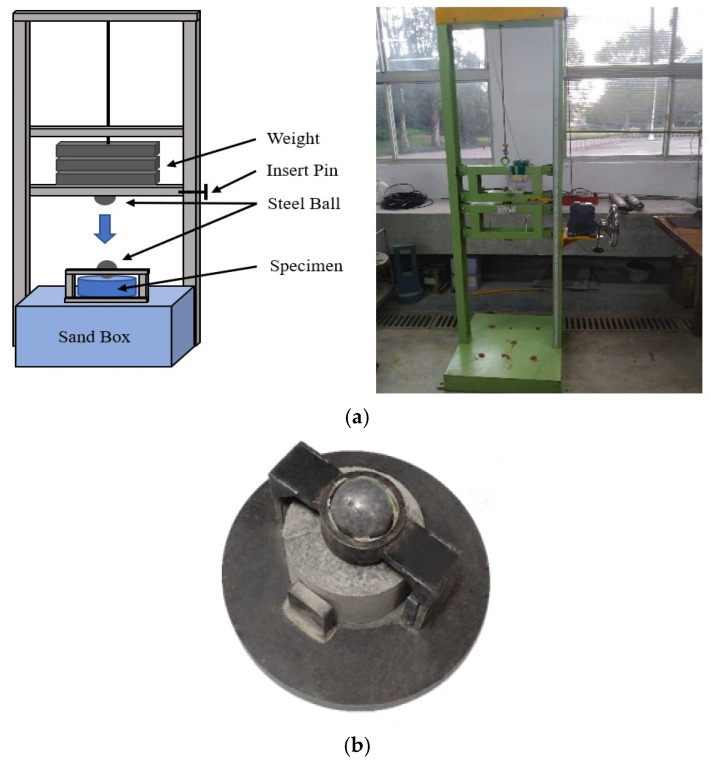
The schematic of impact testing: (**a**) The setup of impact testing; (**b**) The specimen in impact testing.

The fiber-reinforced geopolymer mortar was tested for compressive strength according to ASTM C109 standards. The specimens were situated in a hydraulic machine and subjected to a load speed of roughly 900 to 1800 N/s to determine each specimen’s maximum compressive strength [26]. For these compressive tests, the geopolymer mortar specimens were designed as 5 × 5 × 5 cm cubes. Moreover, the flexural testing was conducted following the ASTM C293 standards to determine the modulus of rupture of the concrete [27]. This method is suitable for specimens having a cross-sectional area of less than 15 × 15 cm. The specimens of the fiber-reinforced geopolymer mortar were designed as 5 × 5 × 20 cm square columns. Following the ASTM D5628 standards [28,29], an impact test was conducted as illustrated in Figure 2. The purpose of this test was to measure the impact energy of a load on a flat cylindrical specimen with a diameter of 15.2 cm and a thickness of 6.35 cm. Furthermore, the force applied to the specimen and which resulted from a specific impact energy was recorded using the force sensor (model: PCB203B).

#### 2.2.2. Experimental Setup and Measurement

**Figure 3:**
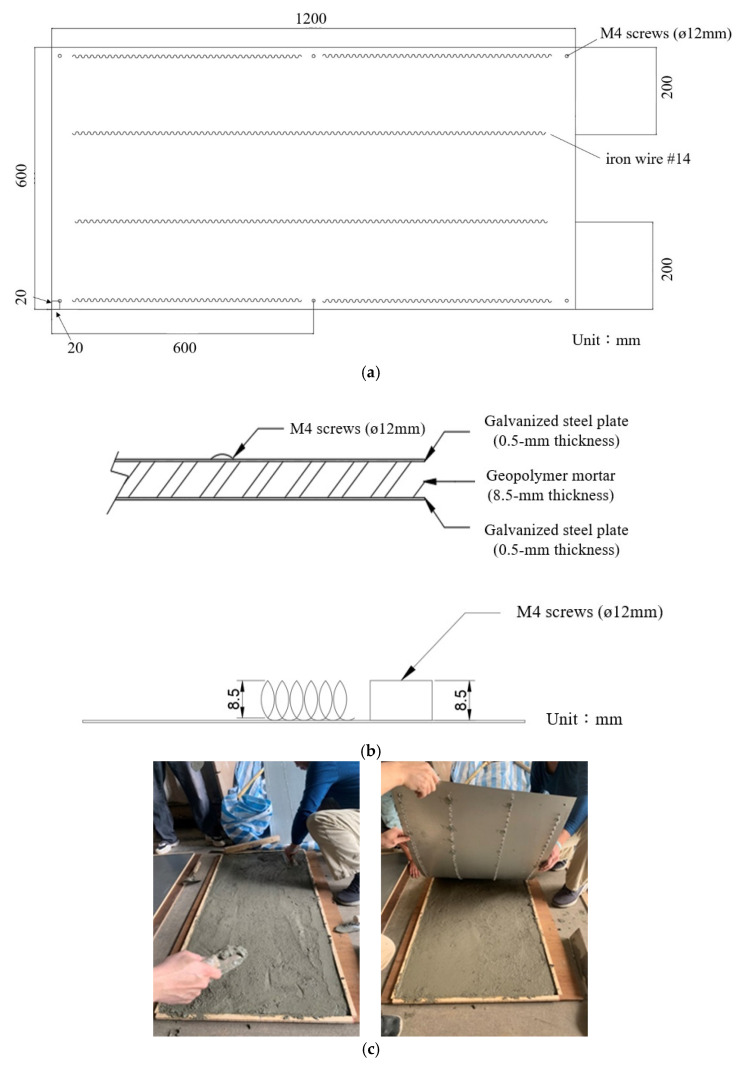

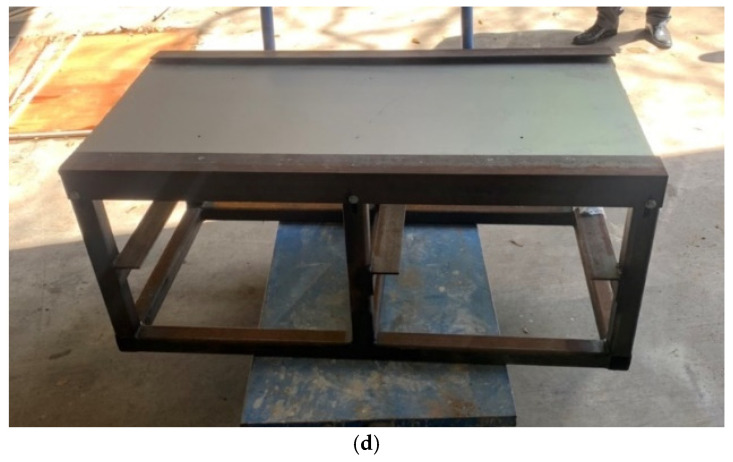
The schematic of the blast resistant panel: (**a**) The blast resistant panel on horizontal view; (**b**) The blast resistant panel on lateral view; (**c**) The manufacture of the blast resistant panel; (**d**) The blast resistant panel.

In the field explosion test, the blast resistant panel was set up in the sandwich structure format. The composite panel consisted of outer layers made of galvanized steel plate (600 × 1200 × 0.5 mm) and an inner layer comprising iron wire embedded with geopolymer mortar (600 × 1200 × 8.5 mm) resulting in a total thickness of 9.5 mm, as depicted in Figure 3a. Figure 3b illustrates a sectional view of the composite panel. The design involves utilizing a spiral-shaped iron wire #14 (ø2 mm), coated with enamel, to connect the upper and lower galvanized steel plates at 200 mm intervals. This arrangement significantly improves the bonding force between the steel and the matrix, as visually depicted in Figure 3. To secure the panel at its longer edge, M4 screws (ø12 mm) were employed. Notably, the steel plates exhibit a yield strength of 200 GPa and an elastic modulus of 210 MPa, respectively. Figure 3c presents the manufacturing process of the blast resistant panel.

**Figure 4:**
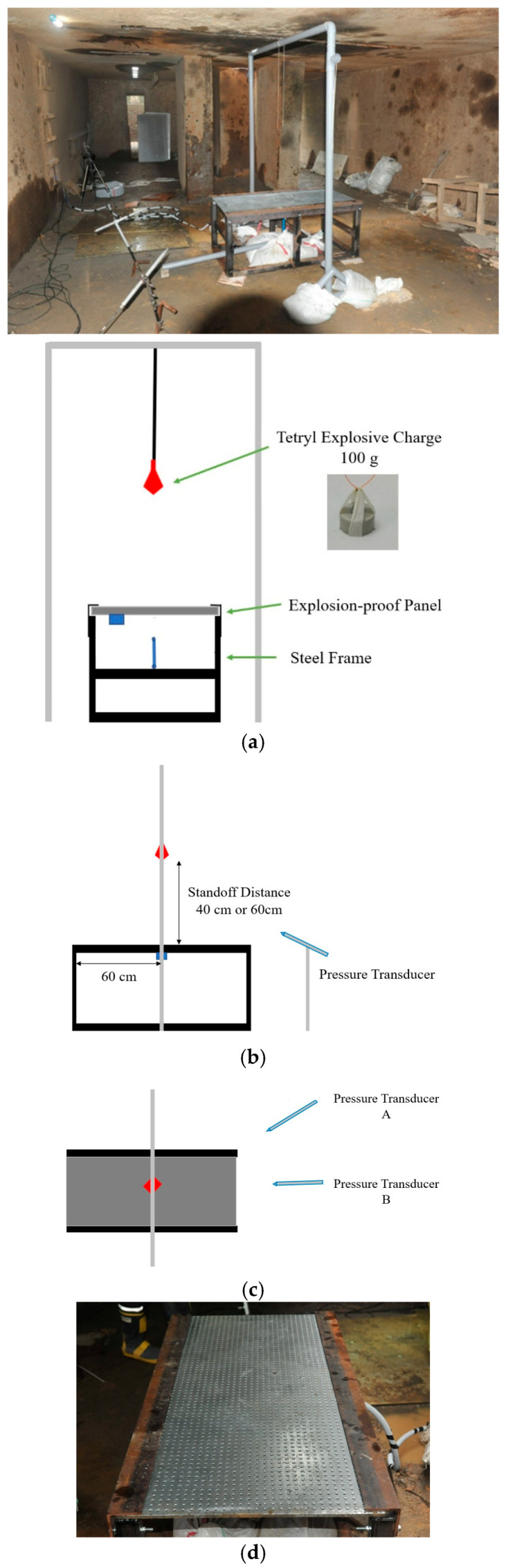
The setup of blast testing: (**a**) Testing arrangement; (**b**) Lateral view; (**c**) Top view; (**d**) Testing specimen.

The schematic of the blast testing is shown in Figure 4. In the configuration, the blast resistant panel was positioned on top of the steel frame. The explosive charge, acting as a free-air explosion, was placed at standoff distances of 40 cm and 60 cm from the center of the upper surface of the panel, respectively. In the experimental program, an explosive charge of Tetryl was utilized, which is a nitramine booster explosive charge with a detonation velocity of approximately 7910 m/s and a density of 1730 kg/m^3^. Furthermore, to measure the overpressure throughout the testing, pressure transducers (137A21 and 137A23, by PCB Piezotronics, Inc., Depew, NY, USA) were set up. These were positioned at closer distances of 133 cm and 142 cm, as well as at farther distances of 160 cm and 170 cm from the center of the top slab, respectively. The explosive phenomenon is caused due to the quick and stable chemical reaction that initiates a detonation wave. The wave rapidly transforms solid or liquid explosive substances into gas that is exceptionally dense, hot, and under high pressure. This explosive gas then serves as the source of intense blast waves in the surrounding atmosphere. In this scenario, the explosion generates blast waves, essentially forming a shock wave characterized by an intense shock front. Typically, the primary blast data outlined in the manual are derived from a bare spherical *TNT* explosive and can be extended to other explosive charges. Using UFC 3-340-02 [22], the *TNT* equivalent mass factor can be presented as the following equation.

(1) $$W_{e}=\frac{H_{\exp}^{d}}{H_{TNT}^{d}}\times W_{\exp}$$

Here, $W_{\exp}$ is the weight of the explosive charge to be converted, $H_{TNT}^{d}$ is the heat of detonation of the explosive charge to be converted, and $H_{TNT}^{d}$ is the heat of detonation of *TNT*. In this study, Tetryl was used as an explosive charge with a *TNT*-equivalent mass factor of 1.07. In the period of explosion, the sudden release of energy resulting from detonation converts the explosive material into a high-pressure gas at extremely high temperatures. The shock wave front is characterized by an instantaneous increase from ambient pressure to peak incident pressure. After reaching the pressure peak, the incident pressure reduces to the pressure lower than the ambient pressure, which is known as the negative phase duration. Since the negative phase duration during explosion has less of an effect on structures, it is generally disregarded in most analysis and evaluation. Therefore, the shape of a shock wave, which includes both the positive and negative phase durations, can be defined by the Friedlander equation, as demonstrated in the following formula.

In a field explosion test, the shock wave front is characterized by an instantaneous rise from the ambient pressure to a peak incident pressure. Once the pressure peak is reached, the incident pressure decreases to a value lower than the ambient pressure, known as the negative phase duration. The configuration of the blast wave in the empirical model can be characterized using the modified Friedlander equation [30], as depicted in the following equation. The ambient pressure and overpressure can be denoted as $p_{0}$ and $p_{pos}$, respectively. The time beginning at the arrival of the pressure wavefront and the duration of positive overpressure can be represented as *t* and $t_{pos}$, respectively. Additionally, the decay coefficient is denoted as *b*.

(2) $$p(t)=p_{0}+p_{pos}\left(1-\frac{t}{t_{pos}}\right)e^{-b\frac{t}{t_{pos}}}$$

In the CONWEP model, the blast pressure at the segment centroid of an element can be modified by the angle of incidence. Consequently, the pressure can be transformed into forces that are exerted on the element. Typically, as a shock wave travels through air and encounters a rigid surface, the wave’s motion is terminated and a new reflected wave is generated, moving in the opposite direction. Considering the angle of incidence, the blast pressure can be modified as effective pressure using Equation (3). When the shock wave approaches at an angle of zero degrees, the effective pressure $p_{eff}$ is equal to the reflected pressure. However, if the shock wave strikes the target surface at an oblique angle, the effective pressure can be modified [31].

(3) $$p_{eff}=p_{reflected}\cos^{2}\theta +p_{incident}\left(1+\cos\theta -2\cos^{2}\theta \right)$$

Regardless of incident or reflected pressure, both pressures can be described as the decay behavior of the pressure using the Friedlander equation as shown below. In the CONWEP model, the blast pressure can be specified using the *BLAST_ENHANCED keyword, while the *BLAST_SEGMENT keyword is used to define the element where the blast load is applied.

(4) $$p_{incident}^{\prime }(t)=p_{incident}(t)\left(1-\frac{t}{t_{pos}}\right)e^{-b_{1}\frac{t}{t_{pos}}}$$

(5) $$p_{eff}=p_{reflected}\cos^{2}\theta +p_{incident}\left(1+\cos\theta -2\cos^{2}\theta \right)$$

In the empirical model, the peak positive overpressure can be estimated through the scaled distance *Z* shown below. In the equation, *R* represents the standoff distance, while *W* represents the weight of the explosive charge.

(6) $$Z=\frac{R}{W^{\frac{1}{3}}}$$

The explosive charge weight of 100 g is positioned at the standoff distances of 40 cm and 60 cm. Thus, the design scaled distances in the field explosion test are 0.862 m/kg^1/3^ and 1.292 m/kg^1/3^, respectively. The panel is clamped along with the long side of it, and an LVDT is positioned underneath the center of the panel to measure the deflection. The field explosion test setup is shown in Figure 4.

## 3. Experimental Results Discussion

### 3.1. Geopolymer Mechanical Performance

**Figure 5:**
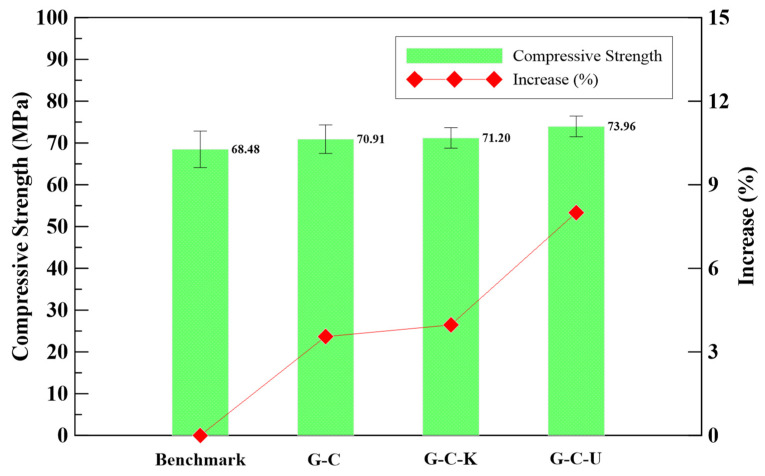
The compressive strength of the fiber-reinforced geopolymer mortar.

The outcomes of the quasi-static compressive strength testing conducted on fiber-reinforced geopolymer mortar according to ASTM C109 standards for 7 days, is illustrated in Figure 5. The average quasi-static compressive strength of geopolymer mortar was 68.48 MPa. Compared to benchmark specimens, the strength of fiber-reinforced geopolymer mortar comprising carbon fiber with a weight ratio of 1.0% was 70.91 MPa, with an increase of 3.6%. Furthermore, the strength of fiber-reinforced specimens composed of a weight ratio of 0.5% carbon fiber and 0.5% Kevlar fiber was 71.20 MPa, representing an increase of 4.0%. Similarly, the specimens which added a weight ratio of 0.5% carbon fiber and 0.5% ultra-high performance PE fiber exhibited a strength of 73.96 MPa, representing an increase of 8.0%. Therefore, for all types of fiber-reinforced geopolymer mortar, an improvement in compressive strength was observed at the age of 7 days.

Based on the physical properties of the fibers used in this study, carbon fiber possesses better tensile strength, which means a better compressive performance can be expected. However, PE fiber has the lowest density, meaning more fibers are distributed in the specimen, which is beneficial for compressive strength growth. This study was designed to replace 50% weight of 1 g carbon fiber with Kevlar and PE fibers for their better breaking elongation. Due to the fiber types’ difference in density, having more fibers distributed in the specimens leads to a better compressive strength, which agrees with the results.

**Figure 6:**
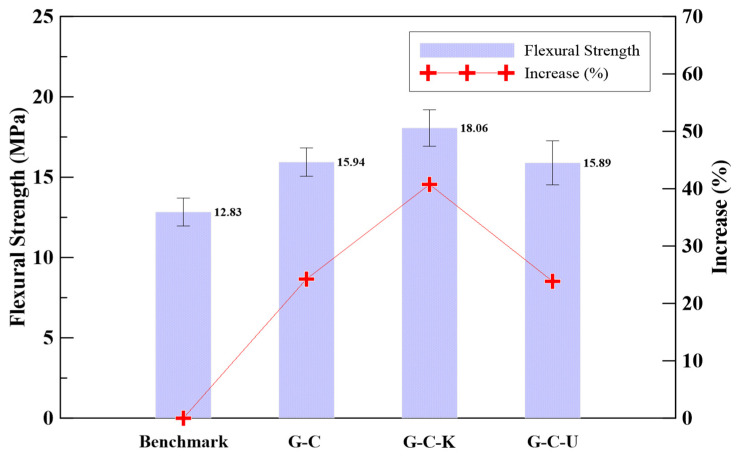
The flexural strength of the fiber-reinforced geopolymer mortar.

The flexural strengths at the age of 7 days of different fiber-reinforced geopolymer mortar specimens were obtained following ASTM C293 standards, and the results are shown in Figure 6. The geopolymer mortar exhibited an average flexural strength of 12.83 MPa. In comparison with the benchmark specimens, the inclusion of carbon fiber at a weight ratio of 1.0% in the fiber-reinforced geopolymer mortar resulted in a strength of 15.94 MPa, increasing by 24.2%. Moreover, the specimens reinforced with a weight ratio of 0.5% carbon fiber and 0.5% Kevlar fiber exhibited a strength of 18.06 MPa, representing a significant increase of 40.7%. Similarly, the specimens incorporating a weight ratio of 0.5% carbon fiber and 0.5% ultra-high performance PE fiber displayed a strength of 15.89 MPa, resulting in a notable increase of 23.9%. Consequently, all types of fiber-reinforced geopolymer mortar exhibited improved flexural strength at the age of 7 days. The results show that G-C-K has better flexural strength. It is noted that in the flexural strength test the tensile strength of the fiber plays an important role. Although there are more fibers distributed in the G-C-U specimen due to its lowest density, the weaker tensile strength of PE fiber led to a lower flexural strength compared to the others.

With an impact energy of 125 J, the geopolymer mortar specimens exhibited the ability to endure a single impact. However, when carbon fiber was incorporated at a weight ratio of 1.0%, the specimens demonstrated an average resistance to 22.0 impacts. The specimens composed of a weight ratio of 0.5% carbon fiber and 0.5% Kevlar fiber exhibited an average resistance to 47.0 impacts. Similarly, the specimens incorporating a weight ratio of 0.5% carbon fiber and 0.5% ultra-high performance PE fiber exhibited remarkable impact resistance, with an average resistance to 251.5 impacts.

**Figure 7:**
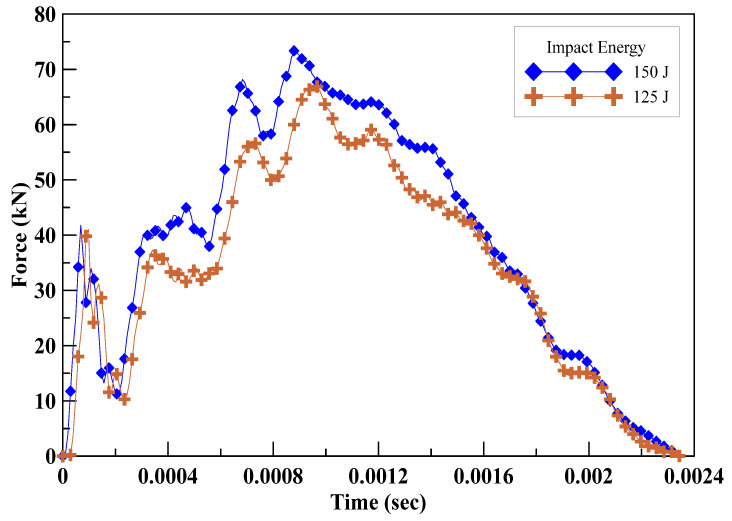
The force applied on the specimens in impact testing.

**Figure 8:**
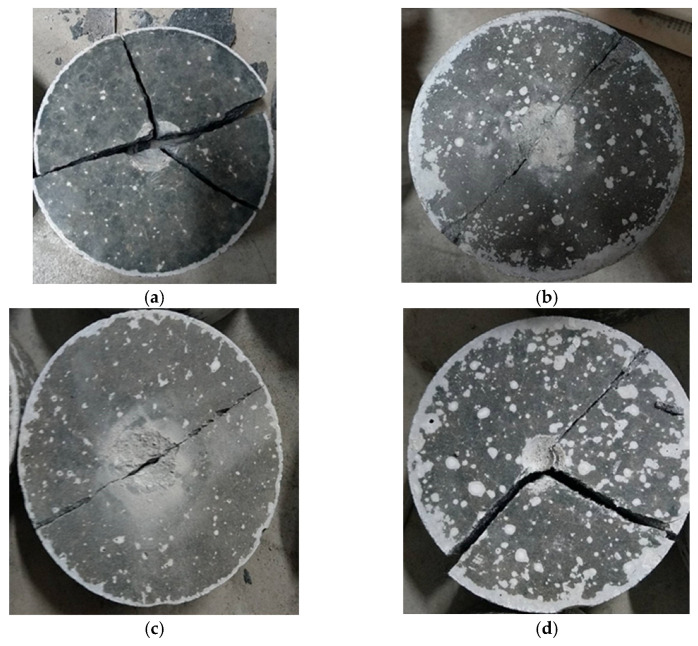
The failure mode observed on the specimens after the impact testing: (**a**) Benchmark; (**b**) G-C; (**c**) G-C-K; (**d**) G-C-U.

When subjected to an impact energy of 150 J, the geopolymer mortar specimens exhibited the capability to withstand a single impact. The specimens reinforced with a weight ratio of 1.0% carbon fiber demonstrated an average resistance to 5.0 impacts. The specimens comprising a weight ratio of 0.5% carbon fiber and 0.5% Kevlar fiber exhibited an average resistance to 2.5 impacts. Similarly, the specimens incorporating a weight ratio of 0.5% carbon fiber and 0.5% ultra-high performance PE fiber showcased significant impact resistance, with an average resistance to 5.0 impacts. As shown in Table 5, the specimens reinforced with a combination of carbon fiber and ultra-high performance PE fiber exhibited superior impact resistance compared to the other specimens. Moreover, the force resulting from the impact was measured using a force sensor, as illustrated in Figure 7. Furthermore, Figure 8 displays the failure mode observed on the specimens after the impact testing. The types of fibers used in this research are all alkali-resistant materials and are all expected to effectively play their role of a bridging effect. Fibers of this kind, when added to geopolymer materials, can effectively prevent the expansion of cracks in the specimens after being subjected to stress due to their ability to absorb and disperse stresses within. Moreover, these fibers can form a network structure in the specimen, restraining the free propagation of cracks and thereby enhancing the crack resistance properties [32,33,34].

### 3.2. Experimental Results

**Figure 9:**
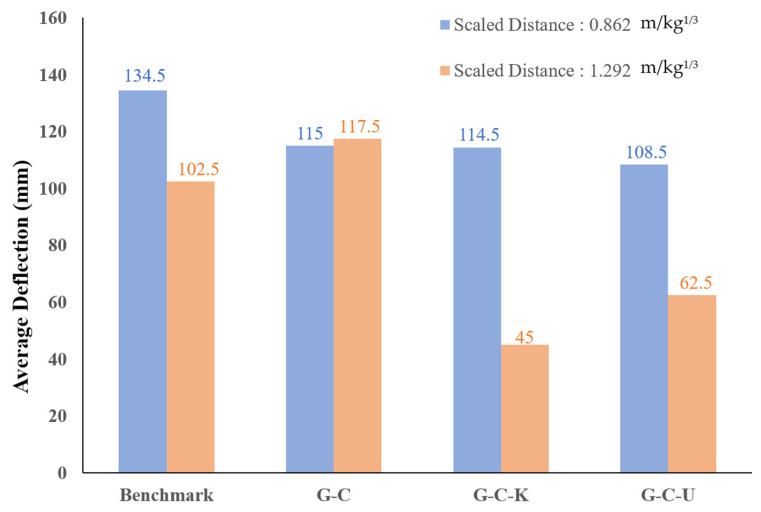
Field explosion test results of the panels under different scaled distances.

**Figure 10:**
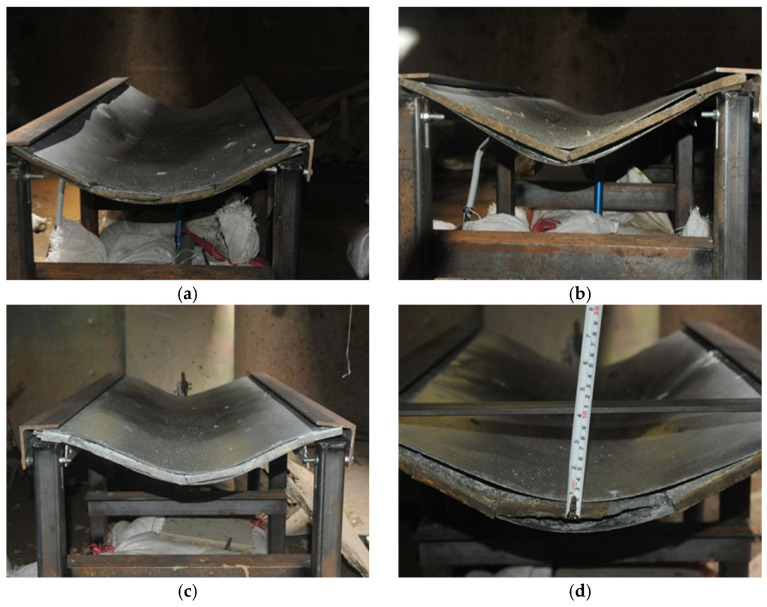
Experimental observation of panels under the 0.862 m/kg^1/3^ blast loading: (**a**) Benchmark; (**b**) G-C; (**c**) G-C-K; (**d**) G-C-U.

**Figure 11:**
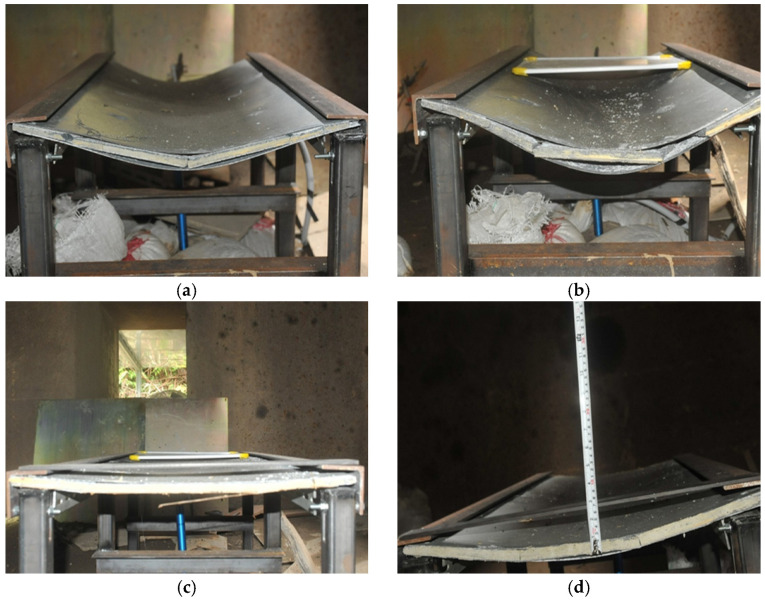
Experimental observation of panels under the 1.292 m/kg^1/3^ blast loading: (**a**) Benchmark; (**b**) G-C; (**c**) G-C-K; (**d**) G-C-U.

Table 6 displays the weight of the explosive charge and the standoff distance for each panel involved in the experimental procedure, whereas Figure 9 provides a summary of the permanent deflection observed in each panel. Since obtaining measurements of midspan deformation was difficult during the experimental procedure, the deflections at both endpoints of the panel were employed. In addition, the experimental observation of specimens under the scaled distance 0.862 m/kg^1/3^ and 1.292 m/kg^1/3^ gives the results in Figure 10 and Figure 11, respectively.

At a scaled distance of 0.862 m/kg^1/3^, the average deflection of the geopolymer mortar was 134.5 mm. When compared to the benchmark specimens, the specimens reinforced with a weight ratio of 1.0% carbon fiber exhibited an average deformation of 115.0 mm, resulting in a decrease of 14.5% in deformation. The specimens composed of a weight ratio of 0.5% carbon fiber and 0.5% Kevlar fiber demonstrated an average deformation of 114.5 mm, decreasing 14.9% in deformation. Similarly, the specimens incorporating a weight ratio of 0.5% carbon fiber and 0.5% ultra-high performance PE fiber exhibited an average deformation of 108.5 mm, decreasing 19.3% in deformation.

At a scaled distance of 1.292 m/kg^1/3^, the average deflection of the geopolymer mortar was 102.5 mm. However, compared to the benchmark specimens, the specimens reinforced with a weight ratio of 1.0% carbon fiber experienced an average deformation of 117.5 mm, resulting in an increase of 14.6% in deformation. Based on the analysis of the failure mode, it was observed that the larger deformation was attributed to the worse dispersion of fiber in the geopolymer mortar during the mixing process. The specimens incorporating a weight ratio of 0.5% carbon fiber and 0.5% Kevlar fiber exhibited an average deformation of 45.0 mm, decreasing 56.1% in deformation. Similarly, the specimens incorporating a weight ratio of 0.5% carbon fiber and 0.5% ultra-high performance PE fiber experienced an average deformation of 62.5 mm, resulting in a decrease of 39.0% in deformation. In general, the results indicated that the addition of fibers enhanced the explosive resistance capacity of the geopolymer mortar panel. In particular, when different types of fibers were combined, the displacement difference among most fiber-reinforced geopolymer mortar panels was smaller compared to the benchmark specimens.

A geopolymer is a brittle material, and adding fiber can improve its mechanical properties, especially for flexural strength and impact resistance, as shown in Figure 7 and Table 5. These results indicate that adding hybrid fibers to geopolymer can increase its energy absorption capacity and further enhance its explosion resistance performance, and this agrees with the average deflection from field explosion test results, as shown in Figure 9. Among these results, the hybrid panels G-C-K possess better performance in flexural, impact, and explosion tests. Due to the complexity of the blast wave propagation and the failure patterns of the composite geopolymer panel, the blast-resistant performance is contributed by compressive strength, tensile strength, and its impact energy absorption capacity. This study explored how adding hybrid fibers with gradient tensile strength and breaking elongation into a geopolymer can effectively enhance its blast resistance capacity.

## 4. Finite element Analysis

### 4.1. Modelling

**Figure 12:**
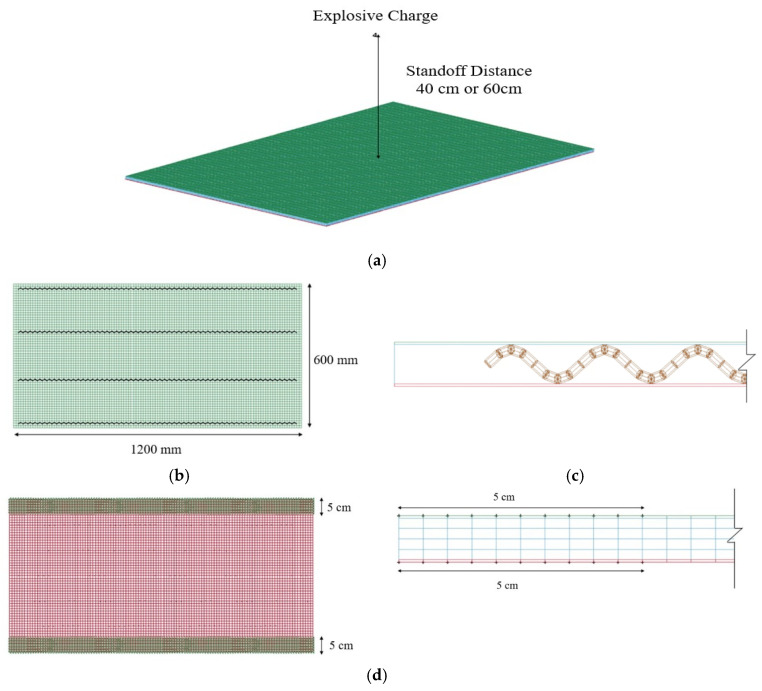
Numerical model in finite element analysis: (**a**) Numerical model; (**b**) Top view; (**c**) Lateral view; (**d**) Support boundary.

To estimate the structural response and potential failure of fiber-reinforced geopolymer mortar panels under dynamic loads characterized by high strain rates, high pressures, and rapid deformations, a 3D finite element model was employed. The model aimed to predict and simulate the damage mechanisms and failure modes of the panels when subjected to close-in blast scenarios. The numerical simulations of the blast experimental procedure were carried out using an explicit finite element code. In this study, the target blast resistant panel has been modeled as a sandwich structure, depicted in Figure 12, comprising a support boundary and an explosive charge. The composite panel was composed of outer layers constructed from galvanized steel plates, while the inner layer consisted of geopolymer mortar embedded with iron wire. The mortar and galvanized steel plates were built using three-dimensional solid elements in the model, while the iron wires were discretized as beam elements. As the iron wires were embedded within the mortar, the interaction was defined through the keywords *CONSTRAINED_BEAN_IN_SOLID. Considering the adhesion of the enamel coating, the contact between the mortar and the galvanized steel plates was modeled using either *CONTACT_AUTOMATIC_NODES_TO_SURFACE or *CONTACT_TIED_NODES_TO_SURFACE keywords. The contact between the nodes and the surface is employed for one-way contact, whereas the TIED contact type is utilized in scenarios where the nodes do not have rotational degrees of freedom. Moreover, considering the interaction between the iron wires and the galvanized steel, the contact was modeled using the *CONTACT_AUTOMATIC_BEAMS_TO_SURFACE keyword, which is appropriate for beam elements.

### 4.2. Material Model

#### 4.2.1. Concrete

Commonly used concrete models in dynamic simulations include the KCC (Karagozian & Case Concrete), Winfrith Concrete, CSCM (Continuous Surface Cap Model), and RHT concrete models. Several strength criteria models exist in mechanics that define the failure surface for concrete, including the Mohr–Coulomb, Drucker–Prager, Ottosen, and Willam–Warnke strength criteria. The KCC and RHT models adopt the Willam–Warnke strength criteria, while the Winfrith Concrete and CSCM models are based on the Ottosen strength criteria.

The continuous surface cap model is a yield criterion that smoothly and continuously combines a shear failure surface with a cap hardening surface through a three-invariant reduction factor on the deviatoric plane shown in Equation (7) [35,36,37].

(7) $$f\left(I_{1},J_{2},J_{3},\kappa \right)=J_{2}-{ℜ}^{2}F_{f}^{2}F_{c}$$

Here, $F_{f}$ presents the shear failure surface, $F_{c}$ presents the cap hardening surface and is a three-invariant factor. In the CSCM model, the shear failure surface on the compressive meridian can be defined in Equation (8), where α, λ, θ, and β are the parameters to fit the shear failure surface in the triaxial compression test. Then, ℜ can determine the strength for any state of stress related to the triaxial compressive strength, where ℜ, a scaling function, changes the radius of the yield surface on the deviatoric plane. The strength surface in triaxial extension or torsion testing can be expressed as $Q_{1}F_{f}(I_{1})$ or $Q_{2}F_{f}(I_{1})$, where $Q_{1}$ and $Q_{2}$ are shown in Equations (9) and (10).

(8) $$F_{f}(I_{1})=\alpha -\lambda e^{-\beta I_{1}}+\theta I_{1}$$

(9) $$Q_{1}=\alpha_{1}-\lambda_{1}e^{-\beta_{1}I_{1}}+\theta_{1}I_{1}$$

(10) $$Q_{2}=\alpha_{2}-\lambda_{2}e^{-\beta_{2}I_{1}}+\theta_{2}I_{1}$$

In the CSCM model, the cap hardening surface can be defined in Equation (11), where κ denotes the variable for cap hardening, X(κ) presents the position where the outer part of the cap intersects on the $I_{1}$ axis, and L(κ) presents a transitional position on the $I_{1}$ axis. L(κ) can be defined as the value shown in Equation (12), and $\kappa_{0}$ denotes the value of $I_{1}$ at the initial intersection of the cap and shear failure surface. When $I_{1}$ is equal to X(κ), the intersection depends on the ellipticity ratio *R* and can be described as Equation (13), where *R* is the ratio of the major to the minor axis. Moreover, the plastic volumetric strain $\varepsilon_{v}^{P}$ can be expressed by Equation (14) based on the motion of the cap in accordance with the hardening rule. Here, W is the maximum plastic volume strain, $D_{1}$ and $D_{2}$ are parameters obtained from fits to the pressure–volumetric strain curves in isotropic compression and uniaxial strain, and $X_{0}$ is the initial position when $\kappa =\kappa_{0}$ [38,39,40,41,42].

(11) $$F_{c}(I_{1},\kappa )=\{\begin{array}{ll} 1-\frac{{\left[I_{1}-L(\kappa )\right]}^{2}}{{\left[X(\kappa )-L(\kappa )\right]}^{2}} & I_{1}>\kappa \\ 1 & otherwise \end{array}$$

(12) $$L(\kappa )=\{\begin{array}{ll} \kappa & if\;\kappa >\kappa_{0}\\ \kappa_{0} & otherwise \end{array}$$

(13) $$X(\kappa )=L(\kappa )+R\cdot F_{f}(\kappa )$$

(14) $$\varepsilon_{v}^{P}=W[1-e^{-D_{1}(X-X_{0})-D_{2}{\left(X-X_{0}\right)}^{2}}]$$

Given the intricate response to brittle materials when subjected to dynamic loads, a series of constitutive material models are available in the finite element analysis software LS-DYNA (Version R11.1.0). Models that are commonly employed in dynamic simulations encompass KCC, Winfrith Concrete, CSCM, and RHT. Among these models, CSCM provides the convenience of requesting default material properties for a comprehensive model description based solely on the unconfined compressive strength and density of concrete-like materials. In the present study, the geopolymer mortar in the blast resistant panels was simulated using the CSCM model. CSCM provides an effective approach to capture the response of geopolymer materials under dynamic loading conditions, making it suitable for accurately representing the behavior of the geopolymer mortar used in the panels. The geopolymer mortar had a mass density of 2320 kg/m^3^, and its unconfined compressive strength was derived from the experimental results presented in this study, as shown in Figure 5.

Moreover, the CSCM model used in this study is based on an elastic–plastic damage model that incorporates the effect of strain rate. It takes into consideration the effective fluidity coefficient to fit the tensile and compressive strain rate data. Given the significant influence of high strain rates in blast testing on geopolymer mortar, the rate effects were enabled in the material model for this study. To assess the damage to geopolymer mortar and to prevent mesh distortion, it is essential to utilize erosion criteria for the elimination of simulation elements that have failed. This is critical as it affects the deformability and crack propagation in simulation. Consequently, an erosion coefficient of 1.05 was defined.

#### 4.2.2. Galvanized Steel Plates and Iron Wires

In the simulation, the *MAT_PLASTIC_KINEMATIC keyword was employed to model the material behavior of galvanized steel plates and iron wires, using an isotropic and kinematic hardening plastic model that takes into account the effects of strain rates. In the simulation, the density and Poisson’s ratio of steel were set at 7850 kg/m^3^ and 0.3, respectively. For the galvanized steel plates, the assigned values for the elastic modulus, yield stress, and failure strain were 210 GPa, 200 MPa, and 0.20, respectively. In the case of the iron wires, the elastic modulus, yield stress, and failure strain were set at 200 GPa, 170 MPa, and 0.15, respectively.

### 4.3. Comparison of Experimental and Numerical Results

#### 4.3.1. Blast Pressure

**Figure 13:**
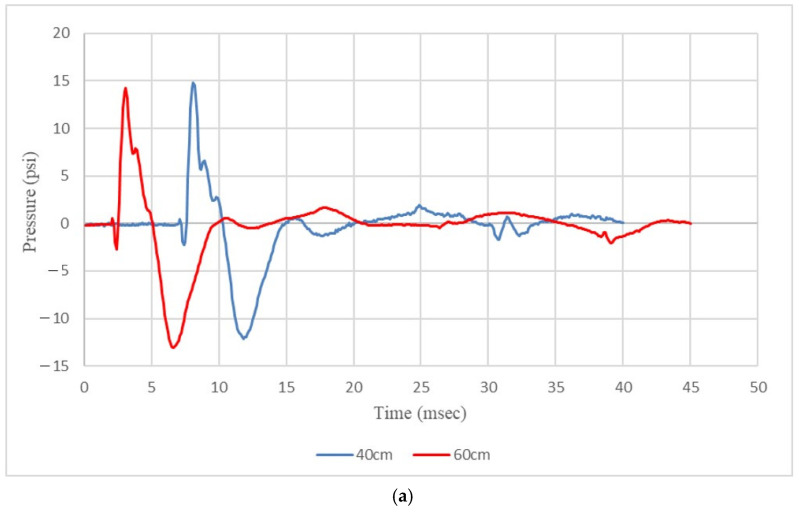

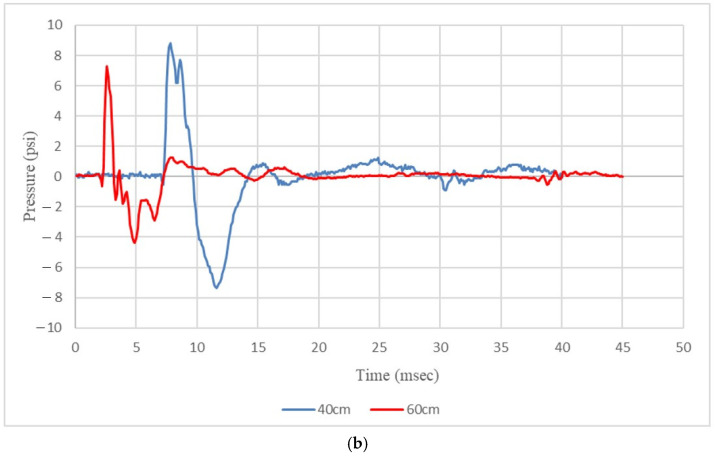
The overpressure measured during the blast testing: (**a**) Pressure transducer positioned at a closer distance; (**b**) Pressure transducer positioned at a farther distance.

**Figure 14:**
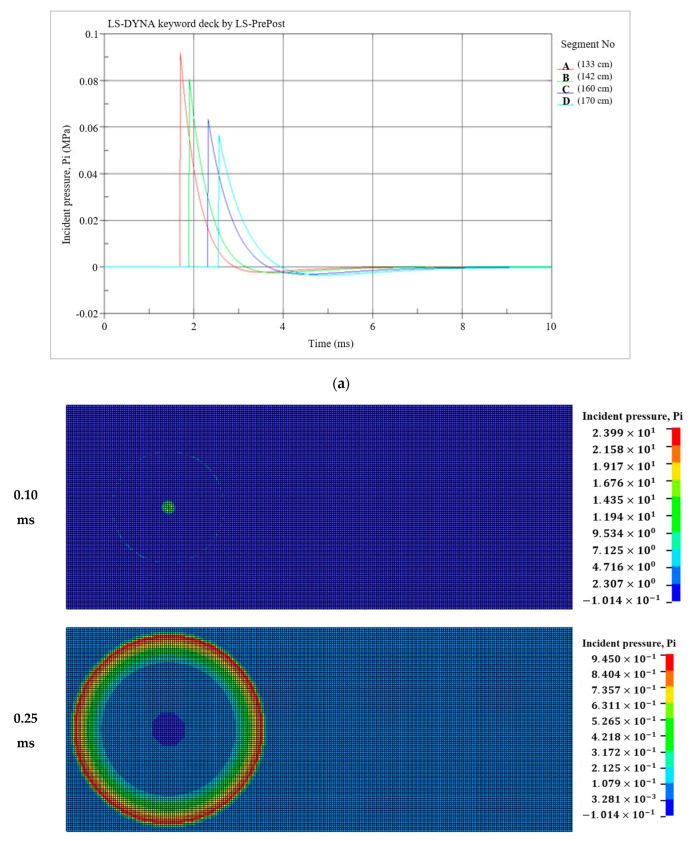

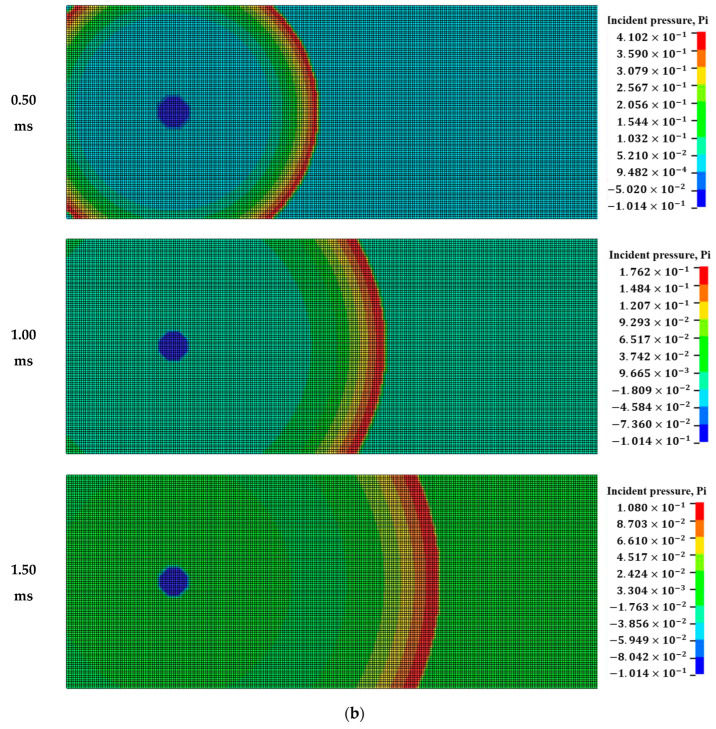
The overpressure and propagation in FE analysis: (**a**) The overpressure in FE analysis; (**b**) The propagation of pressure.

To assess the structural response and potential failure of the fiber-reinforced geopolymer mortar panel during blast testing, a simulated model was utilized. This model analyzed the effects of close-in blasts in free-air and spherical explosions. The overpressure measured by both pressure transducers during the blast testing is depicted in Figure 13. On the other hand, Figure 14 displays the overpressure and its propagation simulated in the finite element analysis.

When the scaled distance was 0.862 m/kg^1/3^ and the pressure transducer was positioned 133 cm from the center of the panel, the overpressures recorded were 0.1018 MPa for experimental results and 0.0919 MPa for numerical results. The results yielded a −9.72% error in peak pressure when comparing both results. Moreover, at the same scaled distance of 0.862 m/kg^1/3^ but with the pressure transducer placed 160 cm from the panel’s center, the overpressures measured were 0.0607 MPa in the experimental results and 0.0634 MPa in the numerical results. In this case, the error in peak pressure between the experimental and numerical results was 4.45%.

When the scaled distance was 1.292 m/kg^1/3^ and the pressure transducer was positioned 142 cm from the center of the panel, the recorded overpressures were 0.0983 MPa for the experimental results and 0.0805 MPa for the numerical results. The results yielded an error of −18.11% in peak pressure. Similarly, at the same scaled distance but with the pressure transducer placed 170 cm from the panel’s center, the measured overpressures were 0.0503 MPa in the experimental results and 0.0565 MPa in the numerical results. The error in peak pressure between the experimental and numerical results in this case was −12.33%.

#### 4.3.2. Deformation of Fiber-Reinforced Geopolymer Mortar

**Figure 15:**
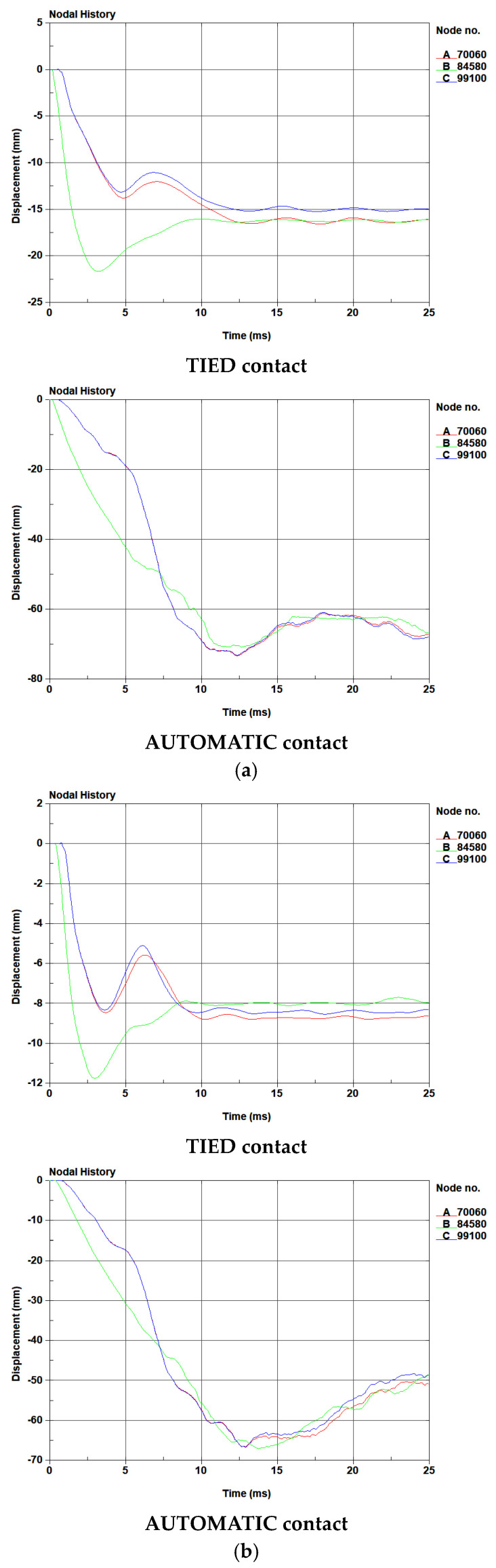
The displacement–time curve of benchmark specimen under different blast loadings simulated in the FE analysis: (**a**) 0.862 m/kg^1/3^ blast loading; (**b**) 1.292 m/kg^1/3^ blast loading.

**Figure 16:**
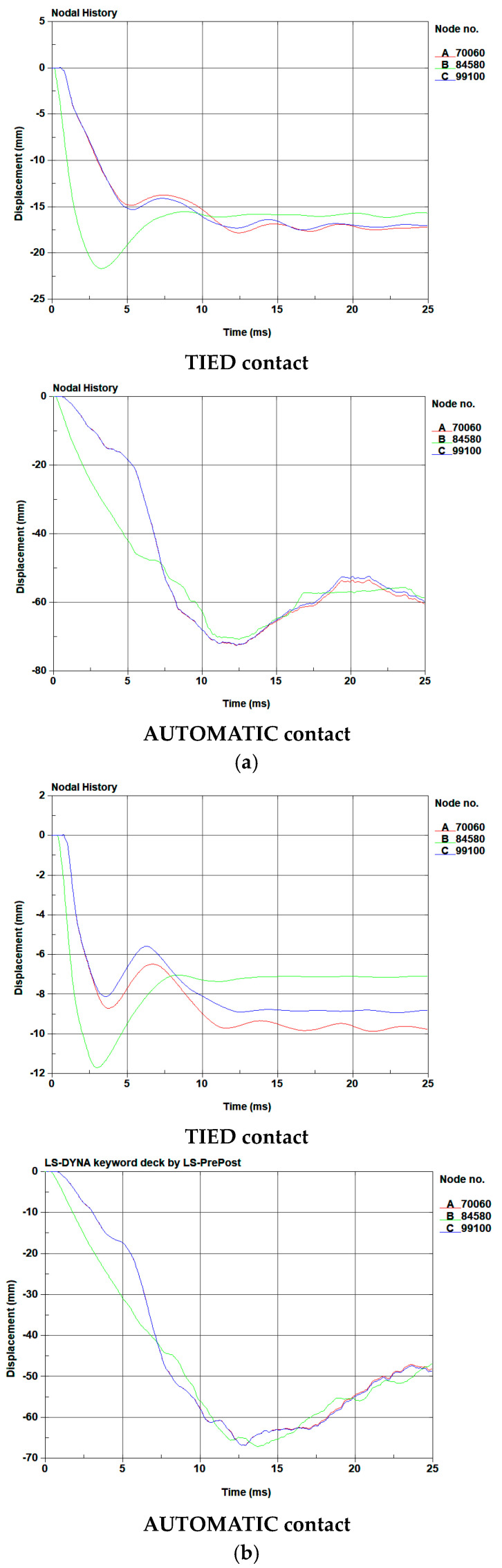
The displacement–time curve of G-C-U specimen under different blast loadings simulated in the FE analysis: (**a**) 0.862 m/kg^1/3^ blast loading; (**b**) 1.292 m/kg^1/3^ blast loading.

In the blast testing, the failure mode of the blast resistant panels was noted to involve a slippage phenomenon occurring at the interface between the mortar and the galvanized steel plates. Consequently, this section investigates the contact at the surface between the mortar and the steel plate, employing the AUTOMATIC and TIED contact keywords in the finite element analysis to compare the results in blast testing. Table 7 presents a summary of the permanent deflection observed in each panel. In the simulation, when employing the TIED contact keyword, the displacement reached an equilibrium state at 15 milliseconds, while using the AUTOMATIC contact keyword led the displacement to reach equilibrium at 25 milliseconds. Consequently, the simulation results for displacement at 25 milliseconds were utilized for comparison with the results from the blast testing. The displacement–time curve, simulated in the finite element analysis, is shown in Figure 15 and Figure 16. Here, the B line demonstrates the midspan deflection of the panel, while the A and C lines represent the deflections at both endpoints of the panel. As measuring the midspan deformation was challenging during the experimental procedure, the deflections at both endpoints of the panel were used instead. In contrast, in the simulation, deformation was evaluated based on deflections at the center point as well as both endpoints of the panel, as depicted in Table 7. Moreover, Table 8 and Table 9 show the numerical failure mode and damage areas of the benchmark specimen in the blast testing.

The permanent deformation at both endpoints of the benchmark panel was measured using a ruler, recording an average of 134.5 mm at a scaled distance of 0.862 m/kg^1/3^. In contrast, in the simulation, the average deflections were notably lower at 15.6 mm and 67.7 mm when employing the TIED and AUTOMATIC contact keywords, respectively. Similarly, for the G-C-U panel, the average permanent deformation measured at both endpoints was 108.5 mm at the same scaled distance. The simulation showed lower average deflections, with values of 17.1 mm and 60.3 mm for the TIED and AUTOMATIC contact keywords, respectively. The discrepancy between the numerical and experimental results for the benchmark panel was 88.4% and 49.7% for the TIED and AUTOMATIC contact keywords, respectively. Similarly, for the G-C-U panel, the difference between the numerical and experimental results was 84.2% and 44.4% for the TIED and AUTOMATIC contact keywords, respectively.

At a scaled distance of 1.292 m/kg^1/3^, the permanent deformation measured at both endpoints of the benchmark panel averaged 102.5 mm. Contrastingly, simulations displayed significantly smaller average deflections of 8.5 mm and 49.9 mm utilizing the TIED and AUTOMATIC contact keywords, respectively. Similarly, for the G-C-U panel, the average permanent deformation measured at both endpoints was 62.5 mm at the same scaled distance. The simulation showed lower average deflections, with values of 9.3 mm and 48.6 mm for the TIED and AUTOMATIC contact keywords, respectively. The disparity between the numerical and experimental data for the benchmark penal was 91.7% and 51.3% when employing the TIED and AUTOMATIC contact keywords respectively. In the case of the G-C-U penal, the difference between the numerical and experimental findings was 85.1% and 22.2% for the TIED and AUTOMATIC contact keywords, respectively.

As depicted in Table 8 and Table 9, using the TIED contact keyword resulted in decreased deformation and the mortar elements remained intact. In contrast, when employing the AUTOMATIC contact keyword, the bottom galvanized steel plate underwent significant deformation, which in turn affected the solid elements of the mortar. Additionally, the absence of constraints in the bond could result in the solid element of mortar failures in the simulation.

Based on both experimental and numerical results, it is evident that the displacements of panels observed in the experimental procedure exceeded those in the numerical results. Notably, when the AUTOMATIC contact keyword was utilized, the outcomes were closer to the experimental results. Taking into account the observations regarding the failure mode of the panels, this discrepancy could be attributed to the worse bonding effectiveness of the coating enamel and screws during the blast testing.

## 5. Conclusions

First, this study investigated enhancing the mechanical properties of geopolymer mortar, including compression strength, flexural strength, and impact resistance, by incorporating various types of fibers such as carbon fiber, Kevlar fiber, and ultra-high performance polyethylene (PE) fiber. In conclusion, based on the mechanical investigation of fiber-reinforced geopolymer mortar, the following conclusions can be drawn.

1. Significant improvements in compressive strength were observed at the age of 7 days for all types of fiber-reinforced geopolymer mortar. Notably, the specimens reinforced with a weight ratio of 0.5% carbon fiber and 0.5% ultra-high performance PE fiber demonstrated a strength of 73.96 MPa, indicating an increase of 8.0%.
2. At the age of 7 days, an enhancement in flexural strength was observed for all types of fiber-reinforced geopolymer mortar. In particular, the specimens reinforced with a weight ratio of 0.5% carbon fiber and 0.5% Kevlar fiber exhibited a remarkable strength of 18.06 MPa, signifying a significant increase of 40.7%.
3. At the age of 7 days, an enhancement in impact resistance was observed for all types of fiber-reinforced geopolymer mortar. In particular, the specimens reinforced with a combination of carbon fiber and ultra-high performance PE fiber exhibited superior impact resistance compared to the other specimens.

Finally, the explosive resistance of the fiber-reinforced geopolymer mortar blast resistant panels was evaluated through the free-air explosion. In this paper, experimental procedures and numerical simulation were performed to study the failure modes, maximum deflection, and dynamic response of the fiber-reinforced geopolymer mortar blast resistant panel under free-air explosion. Some conclusions can be obtained as follows.

1. Overall, the results showed that the incorporation of fibers significantly improved the explosive resistance capacity of the geopolymer mortar blast resistant panel. In particular, when different types of fibers were combined, the displacement difference among most fiber-reinforced geopolymer mortar blast resistant panels was smaller compared to the benchmark panels.
2. From the experimental and numerical data, it is clear that the panel displacements measured in the experiments were larger than those in the simulations. Notably, when the AUTOMATIC contact keyword was employed, the results were closer to the experimental data. Upon considering the failure modes of the panels, this discrepancy can likely be attributed to the worse efficacy of the bond between the coating enamel and screws in the blast testing.

In conclusion, observation of the fiber-reinforced geopolymer mortar blast resistant panels following the explosion test revealed the occurrence of a slippage phenomenon between the outer layer of the galvanized steel plate and the inner layer of the geopolymer mortar. The interlayer shear resistance cannot be provided during the process, so the ability of the composite plate to resist explosion pressure cannot be fully exerted. However, the experimental results showed that the incorporation of fibers significantly improved the explosive resistance capacity of the geopolymer mortar blast resistant panel. Additionally, this study assesses the results obtained from experiments and finite element analysis to serve as a valuable reference for future evaluations of blast resistant performance.

## Figures and Tables

**Table 1:** The chemical composition of geopolymer raw materials determined by XRF.

Chemical Composition (wt. %)	GGBS	Wollastonite	Silica Fume
CaO	57.4	37.5	0.3
SiO_2_	27.4	58.1	96.8
Al_2_O_3_	10.8	2.3	2.2
Fe_2_O_3_	0.6	0.8	-
K_2_O	0.3	-	0.6
TiO_2_	1.2	-	-
L.O.I. *	1.1	-	-
Other	1.2	1.3	0.1

* L.O.I. = Loss on Ignition.

**Table 2:** Physical properties of the fibers.

Specification	Carbon Fiber	Kevlar Fiber	Ultra-High Performance PE Fiber
Density (g/cm^3^)	1.81	1.44	0.97
Tensile Strength (MPa)	3300~6000	2700~3600	1820~3640
Specific Strength (MPa·cm^3^/g)	2750	2080	2680~3600
Tensile Modulus (GPa)	230~550	60~145	79~123
Break Elongation (%)	0.7~2.1	2.4~3.6	3.0~5.0
Deformation Temperature (°C)	1500	450	200

**Table 3:** Abbreviation of the geopolymer material specimens.

Abbreviation	Description
G-B	Geopolymer mortar benchmark
G-C	Geopolymer mortar reinforced with carbon fiber
G-C-K	Geopolymer mortar reinforced with carbon fiber and Kevlar fiber
G-C-U	Geopolymer mortar reinforced with carbon fiber and ultra-high performance PE fiber

**Table 4:** Mix proportion of fiber-reinforced geopolymer specimen.

Mix No.	GGBS	Wollastonite	Silica Fume	Borax	Sand/Binder Ratio	Liquid/Binder Ratio	Fiber		
							Carbon Fiber	Kevlar Fiber	Ultra-High Performance PE Fiber
	(wt.%)	(wt.%)	(wt.%)	(wt.%)			(wt.%)	(wt.%)	(wt.%)
G-B	100	10	1	2	1.2	0.55	0	0	0
G-C							1	0	0
G-C-K							0.5	0.5	0
G-C-U							0.5	0	0.5

**Table 5:** The impact number of the fiber-reinforced geopolymer mortar.

Specimen	Impact Energy (J)	150 J	125 J
Benchmark	Impact number	1	1
		1	1
	Average	1	1
G-C	Impact number	6	23
		4	21
	Average	5	22
G-C-K	Impact number	2	43
		3	51
	Average	2.5	47
G-C-U	Impact number	4	231
		6	272
	Average	5	251.5

**Table 6:** Experimental procedure of blast testing.

Specimen	Standoff Distance (m)	Explosive Charge (kg)	Scaled Distance (m/kg^1/3^)
Benchmark	0.4	0.1	0.862
	0.6		1.292
G-C	0.4		0.862
	0.6		1.292
G-C-K	0.4		0.862
	0.6		1.292
G-C-U	0.4		0.862
	0.6		1.292

**Table 7:** Comparison of experimental and numerical deformation in blast testing.

Specimen	Testing	Simulation							
		TIED Contact				AUTOMATIC Contact			
	Average Deflection (mm)	Average Deflection (mm)	Difference (%)	Midspan Deflection (mm)	Difference (%)	Average Deflection (mm)	Difference (%)	Midspan Deflection (mm)	Difference (%)
Benchmark	134.5	15.6	−88.4%	16.1	−88.0%	67.7	−49.7%	66.9	−50.3%
G-C-U	108.5	17.1	−84.2%	15.7	−85.5%	60.3	−44.4%	58.6	−46.0%
Benchmark	102.5	8.5	−91.7%	8.0	−92.2%	49.9	−51.3%	48.5	−52.7%
G-C-U	62.5	9.3	−85.1%	7.1	−88.6%	48.6	−22.2%	46.9	−25.0%

**Table 8:** Summary of numerical results under 0.862 m/kg^1/3^ blast loading.

Specimen	Contact Keyword	Duration	Short Lateral View	Long Lateral View
Benchmark	TIED	6 ms	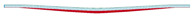	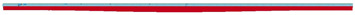
		25 ms	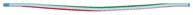	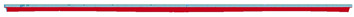
	AUTOMATIC	6 ms	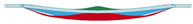	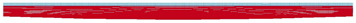
		25 ms	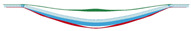	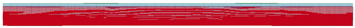
G-C-U	TIED	6 ms	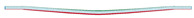	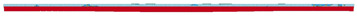
		25 ms	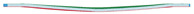	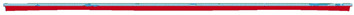
	AUTOMATIC	6 ms	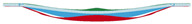	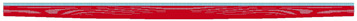
		25 ms	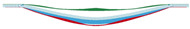	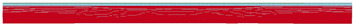

Note: 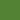 Upper galvanized steel plate; 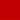 Bottom galvanized steel plate; 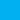 Geopolymer mortar.

**Table 9:** Summary of numerical results under 1.292 m/kg^1/3^ blast loading.

Specimen	Contact Keyword	Duration	Short Lateral View	Long Lateral View
Benchmark	TIED	6 ms	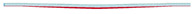	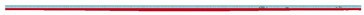
		25 ms	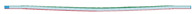	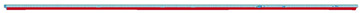
	AUTOMATIC	6 ms	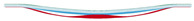	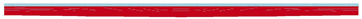
		25 ms	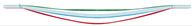	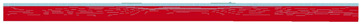
G-C-U	TIED	6 ms	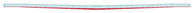	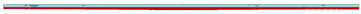
		25 ms	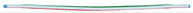	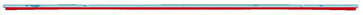
	AUTOMATIC	6 ms	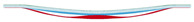	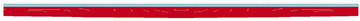
		25 ms	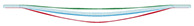	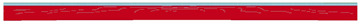

Note: 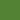 Upper galvanized steel plate; 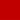 Bottom galvanized steel plate; 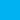 Geopolymer mortar.
